# Rezafungin—Mechanisms of Action, Susceptibility and Resistance: Similarities and Differences with the Other Echinocandins

**DOI:** 10.3390/jof6040262

**Published:** 2020-11-01

**Authors:** Guillermo Garcia-Effron

**Affiliations:** 1 Laboratorio de Micología y Diagnóstico Molecular, Cátedra de Parasitología y Micología, Facultad de Bioquímica y Ciencias Biológicas, Universidad Nacional del Litoral, C.P. 3000 Santa Fe, Argentina; ggarcia@unl.edu.ar or guillermo_garciaeffron@yahoo.com.ar; Tel.: +54-9342-4575209 (ext. 135); 2 Consejo Nacional de Investigaciones Científicas y Tecnológicas, C.P. 3000 Santa Fe, Argentina

**Keywords:** rezafungin, CD101, antifungal susceptibility, mechanism of action, mechanism of resistance, literature review

## Abstract

Rezafungin (formerly CD101) is a new β-glucan synthase inhibitor that is chemically related with anidulafungin. It is considered the first molecule of the new generation of long-acting echinocandins. It has several advantages over the already approved by the Food and Drug Administration (FDA) echinocandins as it has better tissue penetration, better pharmacokinetic/phamacodynamic (PK/PD) pharmacometrics, and a good safety profile. It is much more stable in solution than the older echinocandins, making it more flexible in terms of dosing, storage, and manufacturing. These properties would allow rezafungin to be administered once-weekly (intravenous) and to be potentially administered topically and subcutaneously. In addition, higher dose regimens were tested with no evidence of toxic effect. This will eventually prevent (or reduce) the selection of resistant strains. Rezafungin also has several similarities with older echinocandins as they share the same in vitro behavior (very similar Minimum Inhibitory Concentration required to inhibit the growth of 50% of the isolates (MIC_50_) and half enzyme maximal inhibitory concentration 50% (IC_50_)) and spectrum, the same target, and the same mechanisms of resistance. The selection of *FKS* mutants occurred at similar frequency for rezafungin than for anidulafungin and caspofungin. In this review, rezafungin mechanism of action, target, mechanism of resistance, and in vitro data are described in a comparative manner with the already approved echinocandins.

## 1. Introduction

Over 1 billion individuals are affected by fungal infections worldwide. It is estimated that 11.5 million suffer serious infections and that more than 1.5 million people die from fungal diseases each year [1,2]. Since the beginning of this century, there have been great advances in medical mycology including an increased number of diagnostic tools, the development of reliable methods for assessing antifungal susceptibility, the increased knowledge in mechanisms of antifungal resistance and fungal virulence factors, and the development of new drugs [1,2]. One of the greatest improvements in this last area was the incorporation of echinocandins into the antifungal armamentarium. Until the appearance of this class of drugs, the therapeutic options for invasive mycoses were limited to polyenes, 5-fluorocytocin, and azoles, which have only two molecular targets, the fungal membrane and the synthesis of nucleic acids [3,4].

## 2. β-Glucan Synthase Inhibitors Family Tree

There are different chemical classes of fungal glucan synthesis inhibitors including lipopeptides (pneumocandins and echinocandins), glycolipids (papulocandins) [5], and triterpenoids (ibrexafungerp) [6]. Several compounds were evaluated to establish their antifungal potency and toxicity and even some molecules were subjected to clinical trials. Some of the most promising molecules were arbocandins, papulacandins, enfumagungin, arundifungin, and cilofungin [7,8,9,10,11]. Only three echinocandins are currently in clinical use while the safety and efficacy of rezafungin (echinocandin) and ibrexafungerp (triterpenoid) are being evaluated in different clinical trials [12].

The three FDA-approved echinocandin drugs are semisynthetic derivatives of natural products. In 1974, the first echinocandin-like compound was isolated from *Aspergillus nidulans* var. *echinulatus* [5,6]. It was named echinocandin B and it became the precedent compound for anidulafungin (LY303366) [5,6,7]. Fifteen year later, pneumocandin was isolated from *Lophium arboricola* (homotypic synonym: *Zalerion arboricola*) and it became the compound leading to caspofungin (MK991) [8]. In 1990, three small molecules with antifungal properties were isolated from *Coleophoma empetri* (homotypic synonym: *Thabdostrimina empetri*). Of those, WF11899A side chain was chemically modified to obtain micafungin [13]. Rezafungin (CD101, formerly SP3025, Cidara Therapeutics) is the newest member of the echinocandin family and it is the first member of the second generation of this class of antifungals.

## 3. Chemistry Characteristics and Differences between Rezafungin and the First Generation Echinocandins: Stability, Stability, and Stability

**Figure 1:**
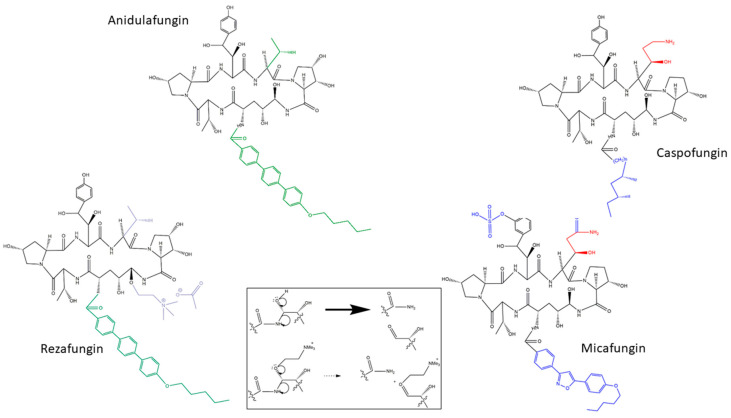
Chemical structure of FDA-approved echinocandins (anidulafungin, caspofungin, and micafungin) and rezafungin. In black, the common chemical structure of the four molecules. In red and green, the caspofungin/micafungin and anidulafungin/rezafungin common structures, respectively. In blue, exclusive micafungin chemical structures. In light blue, the choline aminal ether of rezafungin that improves it stability (degradation less likely). Degradation of anidulafungin and rezafungin, modified from [16].

Lipopeptide antifungals are cyclic depsipeptides (oligopeptides that have at least one of its amide groups, -C(O)NHR-, replaced by its ester, -C(O)OR) with different side chains [14]. Echinocandins are lipopeptides with a cyclic hexapeptide core that contains uncommon amino acids in their structure such as 4-hydroxyproline, 3-hydroxy-4-methilproline, dihydroxyornithine, and dihydroxyhomotyrosine [15]. The depsipeptide core is N-linked to different side chains in each of the drugs. Caspofungin, micafungin, and anidulafungin have a fatty-acyl, an isoxazole 3,5-diphenyl-subtituted, and alkoxytriphenyl side chains, respectively [10,15] (Figure 1). When these side chains are removed by deacetylation, the cyclic core showed no antifungal activity, demonstrating its essentiality for echinocandin activity [6].

First-generation echinocandins share several pharmacological problems. They have poor oral absorption and average half-lives of 14 h (mouse model) [10], making daily intravenous administration mandatory [17,18,19]. These drugs also share chemical instability problems such as thermal and hydrolytic degradation for all three echinocandins [17,18,19] plus photodegradation for micafungin [16,17]. These last inconveniences imply manufacturing and storage troubles and several usage problems including the fact that intravenous preparations have to be used within 24–48 h and that other dosage forms are not possible [16].

Rezafungin is being developed to circumvent many of these pharmacological and stability problems. It is a structural analog of anidulafungin. They share the same side chain and a very similar cyclic core in which the hemiaminal region located at the echinocandin cyclic nucleus (C5 ornithine residue) is replaced with a choline aminal ether (Figure 1). This change reduce the chemical degradation that occurs at the hemiaminal region of anidulafungin, increasing its stability and solubility [16] (Figure 1).

After 44 h of incubation in serum and in phosphate buffered saline (PBS) buffer at 37 °C, more than 94% of the initial amount of rezafungin remains active while these percentages are very low for anidulafungin (10% and 50%, respectively). Moreover, after a year of storage in solution (in saline and in 5% dextrose) with exposure to light, high temperature (40 °C), and acidic conditions and being placed in different buffers there was no (or <7%) decrease in rezafungin antifungal activity, and no epimerization (isomer conversion) was produced. Lyophilisate rezafungin is extremely stable. It only showed less than 2% of degradation products after 9 month storage at 40 °C [16]. These facts are beneficial in terms of dosing flexibility and storage of the pharmaceutical product but also in terms of manufacturing. Moreover, rezafungin has a higher half-life (>130 h, >5 times the half-life of the other echinocandins) [20,21,22]. These properties would allow rezafungin to be administered once-weekly (intravenous) [23] and to develop of new forms of administration such as topical and subcutaneous application. In addition, higher dose regimens were tested and no evidence of toxic effect were noticed in hepatocytes of rats exposed to high doses of rezafungin [24]. Oppositely, using anidulafungin in similar doses and regimen, researchers observed hepatocellular necrosis. This will eventually prevent (or reduce) the selection of resistant strains [21,25].

## 4. Rezafungin and the Older Echinocandin’s Target: The β-Glucan Synthase Complex

**Figure 2:**
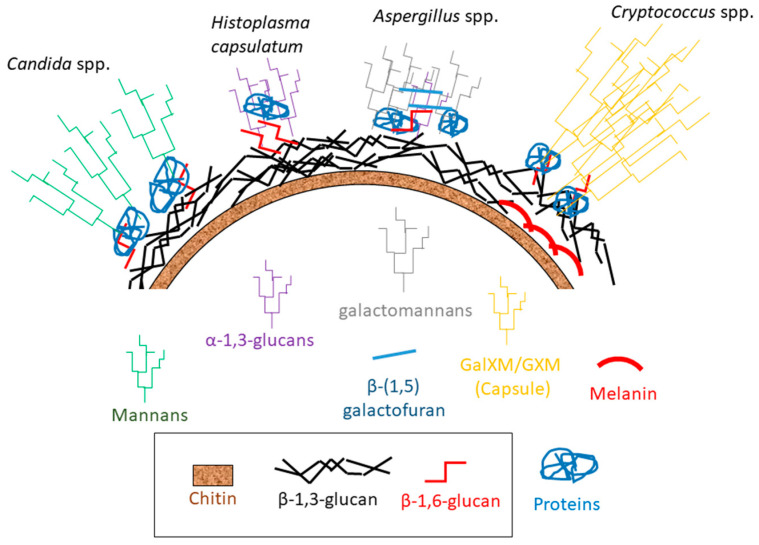
Schematic representation of fungal cell walls of the main fungal human pathogens following the hypothesis proposed by Gow et al. [32]. Alkali-insoluble core components: chithin and branched β-(1,3) and β-(1,6) glucans (black box).

Fungal cell wall is an extracellular matrix composed by an inner layer consisting of carbohydrate polymers and an outer layer of glycoproteins [22,26,27,28]. The main carbohydrates of the fungal cell wall are the β-D-1,3-glucans and α-1,3-glucans accompanied by β-D-1,6-glucans and chitin [6,29,30,31]. These carbohydrates constitute the so-called alkali-insoluble nucleus of the cell wall, which is common for most fungi [32]. On the other hand, the outer layer is substantially different in composition in different fungi. In *Candida albicans*, this layer is mainly formed by mannosylated proteins fixed to the carbohydrate core by glycosylphosphatidylinositol residues [33,34]. In *Aspergillus fumigatus* hyphae (and other filamentous fungi), the mannan chains are shorter than in *Candida* spp. (lower molecular weight), are modified with β-(1,5) galactofuran, and are directly attached to the carbohydrate core [35]. In the Basidiomycete *Cryptococcus* spp., a glucuronoxylomannan-galactoxylomannan capsule is attached to α-1,3-glucans located in the outer cell wall layer while the inner layer is composed of β-D-1,3-glucans, β-D-1,6-glucans, and deacelylated chitin (chitosan) [36,37]. In Ajellomycetaceae such as *Blastomyces dermatitidis* and *Histoplasma capsulatum*, the outer layer of their cell walls is composed by α-(1,3) glucans, which reduce the recognition of the β-(1,3) glucans of the inner layers by immune cells [38,39,40] (Figure 2).

Despite the described differences in composition between species, glucans are the most important structural component of the fungal wall. This carbohydrate alone comprises more than 50% of the dry weight of this structure. Of those glucans, the one with glucose units connected by 1,3 links (β-D-1,3-glucans) represents between 65–90% of the glucan polymers [41]. Responsible for the synthesis of this component of the wall is the 1,3-D-glucan synthase complex (EC 2.4.1.34.). It is a transmembrane protein complex of partially known qualitative and quantitative composition. It is formed by at least two main subunits: Rho1p and Fksp. The former is a regulatory element of the complex [42]. It has promiscuous functions since its participation in several biosynthesis pathways has been demonstrated [43,44,45,46]. Rho1p is known to have Guanosine triphsphatase (GTPase) activity (Ras-like GTP-binding protein) [47,48,49], and as a subunit of the 1,3-β-D-glucan synthase complexes, it is estimated that one of its functions is to provide the necessary energy for the 1,3-β-D-glucan bonds to occur [50]. This idea is supported by the fact that GTP is necessary to produce 1,3-β-D-glucans in vitro using semi-purified 1,3-β-D-glucan synthase complexes [51,52,53]. A second function of Rho1p is to sense stress signals due to 1,3-β-D-glucan depletion and to activate as a response a wide variety of effectors ranging from protein kinase C, *SLT2* [44], calcineurin/*CRZ1*/Ca^2+^, and *HOG* [54,55] to Hsp90p, Mkc1p, and Sgt1p [46,56,57,58,59].

As a complex, 1,3-β-D-glucan synthase is a glucosyltransferase that generate glycosidic bonds (β-1,3 links). It catalyzes the reaction Uridine diphosphate-glucose (UDP-glucose) + {1,3-β-D-glucosyl}_n_ = UDP+ {1,3-β-D-glucosyl}_n+1_, incorporating around 6 nmol UDP-glucose/min with a good affinity for its substrate (average *Km* and *V_max_* for echinocandin susceptible *C. albicans* and *Candida glabrata*: 0.099 ± 0.022 mM and 5.962 ± 0.723 nmol/min and 0.133 ± 0.015 mM and 6.812 ± 0.246 nmol/min, respectively) [51,52]. The Fksp subunits are the real catalyst subunits of the complex and the specific target of echinocandin drugs. This last conclusion is based on the fact that resistance and the subsequent treatment failures is conferred by amino acid substitutions in the Fksp (see below for more details) [60]. Three different homologous genes named *FKS1*, *FKS2*, and *FKS3* encode these proteins. They were originally identified in the 1990s in *Saccharomyces cerevisiae* [61]. Later, *FKS* orthologs were described in several fungal species [53,61,62,63,64,65,66,67] and their particular functions and the way in which their expression is regulated appears to have small differences.

In diploid organisms such as most *Candida* spp., the disruption of both *FKS1* alleles affects cell growth and leads to hypersensitivity to FK-506 (calcineurin phosphatase inhibitor). Thus, *FKS2* is considered a backup gene responsible for producing a cell wall in response to glucose starvation and osmotic stress (as happens when 1,3-β-D-glucans of the wall are reduced) through the calcineurin pathway [68]. On the other hand, *FKS1* expression predominantly occurs during growth on glucose and is constitutively regulated during the cell cycle [68]. The disruption of both alleles of *FKS1* and *FKS2* is lethal [68,69], suggesting that (i) both proteins are component of the complex and (ii) that both subunits are essential and have overlapping function [7,68]. In haploid organisms such as *C. glabrata*, the disruption of *FKS1* and *FKS2* is also lethal but the disruption of *FKS1* and *FKS3* or *FKS2* and *FKS3* is not. Moreover, in this yeast it seems that *FKS1* and *FKS2* are functionally redundant [70]. In the haploid *Aspergillus fumigatus* also, *FKS1* is non-essential and its knockout leads to a compensatory increase of cell wall galactosaminogalactan and chitin and the depletion of galactomannan [50]. Oppositely, in *Yarrowia lipolytica* (heterotypic synonym: *Candida lipolytica*), there is only one *FKS* homologue (*FKS1*) that is essential for viability [62].

This enzyme complex is an ideal antifungal target since it is essential for viability of fungi and it is not present in mammal cells.

## 5. The Place of Rezafungin in the Clinical Setting

Anidulafungin, caspofungin, and micafungin are the drugs of choice to treat *Candida* spp. deep-seated infections [55] while they are recommended as salvage therapy (either alone or in combination with azole drugs) for different types of aspergillosis [71]. Moreover, some reports described the anti-pneumocystis activity of the first-generation echinocandins since they showed the capacity to inhibit cysts but not trophic cells [72]. However, they were not proposed as a choice to treat *Pneumocystis* spp. infections.

Everything seems to indicate that rezafungin share the same spectrum and it will be useful in the same clinical scenarios as the first generation of echinocandins. ReSTORE and STRIVE [73] clinical trials are being carried out to prove the usefulness of rezafungin (both compared with caspofungin) as treatment of candidemia and other invasive candidiasis and as treatment of these mycoses followed by oral step-down treatments. Additionally, rezafungin is also being tested as a therapeutic tool to treat acute and recurrent vaginal yeast infections (RADIANT study) [74] and as a prophylaxis of invasive fungal infections (including *Candida* spp., *Pneumocystis* spp., and *Aspergillus* spp.) in adults undergoing allogenic blood and marrow transplantation (ReSPECT study) [75].

After approval, rezafungin will expand the clinical span of echinocandins as a wider ranging prophylaxis tool and as a way to treat recurrent and refractory vaginal candidiasis.

## 6. Molecular Mechanism of Action of Rezafungin

As mentioned, echinocandins inhibit cell wall formation, specifically 1,3-β-D-glucan synthesis. The consequence of the depletion of 1,3-β-D-glucans in the cell wall are cell morphology changes resulting in osmotic instability and the subsequent cell death and/or inhibition [76]. These antifungals act as fungicidal agents against most *Candida* spp. (such as *C. albicans*, *C. dubliniensis*, *C. krusei*, and *C. tropicalis*) [76,77,78]. For other *Candida* species showing naturally occurring polymorphisms at Fksp that alter its in vitro echinocandin susceptibility (*Candida parapsilosis* complex and *Meyerozima guilliermondii* (heterotypic synonym: *Candida guilliermondii*)) [79,80], these drugs behave differently in vitro, requiring more time and higher doses to reach the fungicidal threshold [81,82], while other emerging species as *Candida auris* are tolerant in vitro to these drugs [83].

In filamentous fungi, echinocandin effects are dependent on the relative cell position within the hyphae. The inhibition of 1,3-β-D-glucan synthase complexes is produced in apical cells, leaving the subapical cells almost intact [13]. This is possible because the cell wall constitutive polymers are in continuous production, modification, and degradation during the cell cycle and hyphal growth. Specifically, it was demonstrated that 1,3-β-D-glucan synthesis is concentrated where the cell wall is remodeling. Fks1p is co-localized with actin patches that are constantly moving on the membrane cell surface to the locations were this polymer is mostly needed [84]. In apical cells, echinocandins produce a misbalance of the glucan content of the wall. They inhibit 1,3-β-D-glucan production, not altering its degradation speed. This fact leads to the bursting of these cells due to osmotic pressure [85]. This kind of apical inhibition of growth is used to establish the minimal effective concentration (MEC) in susceptibility testing assays for filamentous fungi. The MEC is the lowest echinocandin drug concentration that leads to the growth of compact hyphal forms that provides accurate and reproducible susceptibility data [86].

To comprehend the molecular mechanism of action of echinocandins, we need to understand how these cyclic peptides with lipid tails interact with their targets.

**Figure 3:**
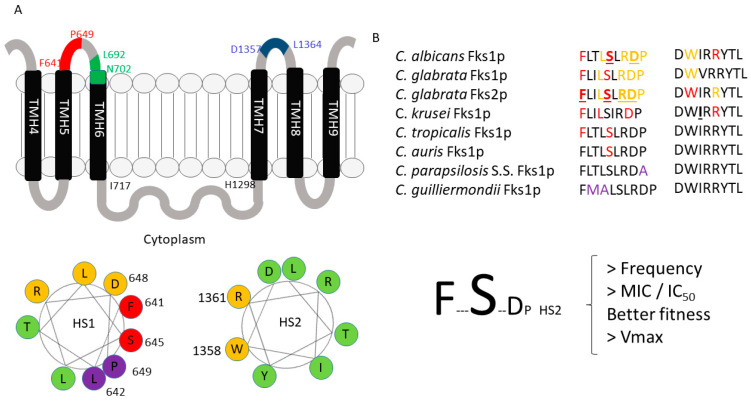
((**A**) above) Schematic representation (not in scale) of a portion of the *Candida albicans* Fks1p membrane topology on the basis of the *Saccharomyces cerevisiae* topology model (98). Black bars represent the transmembrane helices (TMH 4 to THM 9). Hot spot regions are colored in red (hot spot 1), in blue (hot spot 2), and in green (hot spot 3). Hot spot relative positions are not indented to represent the real position or the so-called echinocandin binding pocket. Putative glycosyltranferase domain (catalytic domain and substrate binding sites) is represented by a gray loop (I717-H1298). ((**A**) below) Helical wheel representation showing the putative position of the amino acid of hot spot 1 (HS1) and hot spot 2 (HS2) of *C. albicans* Fks1p arranged in a α-helix seen from above [94]. The residues where mutations confer strong echinocandin resistance phenotype are represented in red; in orange, intermediate to low resistance phenotype [95]; and in purple, the residues that show low phenotype and are implicated in the so-called intrinsic reduced echinocandin susceptibility (IRES) phenotype in *C. parapsilosis* complex (P649A) and *C. guilliermondii* (L642M) [79,80]. The residues not linked with echinocandin resistance are represented in green. ((**B**), above) Alignment of the hot spot regions of *Candida* spp. The residues linked with high level echinocandin resistance are represented in red; in orange, the residues that conferred low to intermediate resistance phenotype [96]; and in purple, the residues confirmed molecularly that are related with IRES phenotype [79,80]. In bold and underlined are the residues where spontaneous mutations were produced after in vitro exposure to high concentrations of rezafungin [97]. ((**B**), below) Simplified representation of the relative frequency of appearance of each substitutions in clinical isolates together with its main characteristics (highest the size, the highest Minimal Inhibitory Concentration (MIC)/ and half enzyme maximal inhibitory concentration 50% (IC_50_), Vmax, and better fitness) [51,52,60,98,99].

Fungal 1,3-β-D-glucan synthase complexes were never fully purified. Some ideas about how this complex works could be glimpsed when semi-purified enzymes (using a methodology called trapping) are studied. Among the data obtained, a factor that stands out is its non-competitive way of inhibition, confirming the inhibition of this complex by echinocandins and some biochemical parameters of the complex as a whole (Ki, Km, and Vmax) [51,52,87]. Thus, we know that these drugs bind to an allosteric site of the enzymes (different from the active site) and that the substrate (UDP-glucose) is located in the cytosolic surface of the membrane and the product (1,3-β-D-glucans) is external [7,61,68]. Additionally, the description of the molecular mechanisms of clinical echinocandin resistance allowed us to have an insight into how these drugs interact with Fksp. These mechanisms are exclusively related to amino acid substitutions in two limited Fksp regions (named hot spot regions 1 and 2). Another hot spot region was suggested and named hot spot 3, which was linked to intrinsic resistance in *Scedosporium* spp. [41,51,52,53,88,89]. These facts demonstrated that echinocandins exert their inhibitory activity by binding to these regions. Efforts to effectively demonstrate where the substrate-binding sites are located were made using *Neurospora crassa* partially purified β-1,3 glucan synthase. The obtained data located this site are in a region of 200 residues (amino acids (aa.) between the residues V1073 and R1277 using *C. albicans* Fks1p numbering) [90]. In 2012, Johnson and Edlind described the Fksp putative substrate and echinocandin binding sites more accurately using *S. cerevisiae* as model organisms [91]. They combined in silico and site-directed mutagenesis analysis together with the analysis of C-terminal fusions (Fks1p-hemagglutinin-Suc2-His4C), followed by His4C expression and Suc2 glycosylation assays [92]. They found that the three described hot spot regions are located in the outer layer of the membrane and are adjacent to each other and that they are external or partially embedded to the plasma membrane. Hot spot regions 1 and 3 are located within transmembrane segments 5 and 6 while hot spot region 2 is located between transmembrane segments 7 and 8. They suggested that they would be possibly three-dimensionally arranged, forming a putative echinocandin-binding pocket, and that echinocandin would be interacting outside the cytoplasm, not requiring the entry into the cell (Figure 3). Additionally, they demonstrated that the glycosyltranferase domain (catalytic domain) should be located in the cytoplasm face of the membrane. It is formed mainly by hydrophilic amino acids (between I715 and H1294 for *S. cerevisiae* Fks1p and amongst the residues I717 and H1298 in *C. albicans* Fks1p) and it is surrounded by the hot spot regions [91] (Figure 3). Following this model, the lipid tail (essential for echinocandin activity) [93] would interact with the partially membrane-embedded hot spot 3 region (L692-N702 in *C. albicans* Fks1p) or near the hot spot 1 region while the cyclic hexapeptide core would do so with hot spot 1 and 2 regions. What has been described indicates that the binding of echinocandin to the three hot spot regions reduces or cancels any glycosyltransferase activity, leading to the depletion of beta glucans and osmotic instability of fungal cells. Considering that (i) the putative localization of the hot spot 1 and 2 regions (opposite to each other), (ii) that hot spot 3 regions bind to the echinocandin tail, (iii) that all three hot spot regions are arranged surrounding the hydrophilic catalytic domain and forming an echinocandin binding pocket, (iv) that echinocandin resistance is almost exclusively linked with mutations in the hot spot 1 and 2 regions, and that (v) hot spot regions are located in the extracellular surface of the membrane, we can speculate that the echinocandin cyclic core is binding or interacting with both hot spot 1 and 2. Moreover, knowing that β-1,3-D glucan synthase substrate (UDP-glucose) is mainly found in the cytosol while the glucan polymer is external, we can also speculate the echinocandins are preventing the exit of the formed polymer by some type of steric hindrance (Figure 3).

## 7. In Vitro Data

The analysis of rezafungin in vitro susceptibility data was performed using a search on pubmed.ncbi.nlm.nih.gov using as keywords “rezafungin” and “CD101 antifungal”. The antifungal susceptibility and surveillance papers were included in the data analysis. Moreover, certain papers with other aims were included, considering the fact that authors published susceptibility data as secondary data or as data that support further studies. Most of the in vitro data were obtained using Clinical and Laboratory Standards Institute (CLSI) reference protocols (documents M27

A3 and M274th ed. for yeasts and M38A2 and M38-3rd ed.) [100,101,102,103,104,105,106,107]. Recently, some differences in rezafungin minimal inhibitory concentration (MIC) were noticed when using the European Committee on Antimicrobial Susceptibility Testing (EUCAST) method and different polystyrene plates were used (culture treated and untreated). MICs values were within three dilutions and untreated polystyrene plates were better to separate wild type and non-wild type strains and also improved the interlaboratory reproducibility [108].

Little difference was seen between reports when MIC/MEC ranges and MIC_50_ and MIC_90_ data were evaluated, despite the used reference method and the different laboratory where the tests were performed (Table S1).

The analyzed papers included susceptibility data of 7818 strains of 17 different *Candida* spp. and 429 isolates of 9 *Aspergillus* spp. (some of the strains were identified at the section level). In one of the reports, 44 strains of *Cryptococcus neoformans* var. *grubii* were studied. All the strains showed very high MIC values (>8 µg/mL), confirming the well-known inactivity of echinocandins in Basidiomycetes [109]. Despite the variety of species included, the published data are focused mainly in the most common *Candida* spp. (*C. albicans*, *C. glabrata*, *C. krusei*, *C. parapsilosis*, and *C. tropicalis*), *C. dubliniensis*, and *C. auris* (Table 1). The in vitro rezafungin susceptibility was studied in only 61 echinocandin-resistant strains (*FKS* mutants). Out of these mutants, the vast majority were *C. glabrata* and *C. albicans* (as occurs in clinical settings) (Table 2).

Rezafungin MICs were very similar to the values obtained for the other echinocandins used as comparators. When MIC_50_ ratios were analyzed (Rezafungin MIC_50_/comparator MIC_50_), small differences were found, especially when compared with anidulafungin MIC_50_. For *C. albicans* and *C. tropicalis*, rezafungin MIC_50_ values were slightly higher than those for anifulafungin (rezafungin MIC_50_/anidulafungin MIC_50_: 2.26- and 2.17-fold, respectively), lower for caspofungin (0.38- and 0.65-fold, respectively), and similar for micafungin (1.56- and 1.39-fold, respectively). For *C. glabrata*, *C. krusei*, and *C. parapsilosis sensu stricto*, MIC_50_ values were almost equal for rezafungin than for anidulafungin (rezafungin MIC_50_/anidulafungin MIC_50_: 1.03-, 0.9-, and 0.64- fold, respectively). Rezafungin seems more potent in vitro than caspofungin and micafungin against *C. krusei* (MIC_50_ ratios: 0.12 and 0.31, respectively), while caspofungin was more potent than rezafungin when *C. parapsilosis* was tested (rezafungin MIC_50_/caspofungin MIC_50_ ratio: 2.58).

As with the other echinocandins, *Candida* spp. showing intrinsic reduced echinocandin susceptibility (IRES) phenotype (*C. parapsilosis sensu lato*, *C. guilliermondii*, *C. lusitaniae*, etc.) showed 10- to 50-fold higher MIC_50_ than those of *C. albicans* (e.g., rezafungin MIC_50_ geometric means of 1.12 vs. 0.023 for *C. parapsilosis sensu stricto* and *C. albicans*, respectively) (Table 1).

Rezafungin was more potent than caspofungin and micafungin against *C. auris* (rezafungin MIC_50_/comparator MIC_50_ ratios = 0.31 and 0.65, respectively). On the other hand, rezafungin MIC_50_s were twofold higher than those for anidulafungin (MIC_50_ ratio 2.55) (Table 1). Rezafungin, anidulafungin, caspofungin, and micafungin MIC values for *C. auris* were 6.57, 5.81, 8.24, and 16.7 times higher than for *C. albicans*, respectively.

Rezafungin shares similar potency against *FKS* mutants than the other echinocandins. The MIC values for mutants were between 6 to 50 times higher for mutants than for those of wild type strains of the same species (Table 2). The highest MIC differences between mutants and wild type strains were observed in *C. auris FKS* mutants. For this species, only four mutants harboring the same mutation (S6639P equivalent to S645P for *C. albicans*) were studied. This amino acid substitutions are responsible for the most prominent resistance phenotype in all *Candida* spp. [60].

Turning to *Aspergillus* spp., rezafungin was very potent for all the studied species, even for cryptic multi-resistant species of the *Aspergillus* section Fumigatii. For these intrinsically azole-resistant species and for secondary resistant *Aspergillus fumigatus sensu stricto*, rezafungin would be a good treatment option, as has been suggested for the other echinocandins (treatment of azole refractory aspergillosis) [71] (Table 3, with more detail in Table S1).

There were other reports that were not included in the initial analysis since they used different antifungal susceptibility testing methods, but it is nonetheless important to analyze them due to the potential use of rezafungin as treatment of vulvovaginal candidiasis (topical formulation) and to act in biofilms. Rezafungin susceptibility of *Candida* species isolated from patients with acute and recurrent vulvovaginal candidiasis was evaluated using a modified CLSI method (performed at pH = 4 to resemble vaginal pH) [112]. Authors found that rezafungin showed a potent activity against *C. albicans*, *C. glabrata*, *C. parapsilosis*, and *C. tropicalis* isolated from vulvovaginal infections. Moreover, these MIC values were similar to those obtained for *Candida* spp. isolated from deep-seated infections [102,103,104]. The obtained MICs (even for *C. parapsilosis*) were below the intravaginal rezafungin concentration that would be reached after topical administration [112]. To evaluate the activity of rezafungin against *C. albicans* biofilms, Chandra et al. studied the metabolic activity of the cells in early and mature biofilms (formed on silicone elastomer discs) and the thickness of the formed structures by confocal microscopy [113]. When rezafungin was used at 0.25–1.00 µg/mL, the authors observed a reduction in biofilm thickness (in both early and mature preformed biofilms). In addition, at the same drug concentration, the development of mature biofilms was prevented [113].

## 8. Mechanisms of Resistance

As with any antibiotic, microbiological resistance to echinocandin drugs can be divided into innate or inherent and secondary or acquired resistance. The first definition includes species that show non-wild type susceptibility patterns of all or almost all strains (susceptibility testing is unnecessary to establish its resistance) [114]. The former designation includes strains that obtain the ability to resist the activity of a particular antimicrobial agent to which it was previously susceptible [115]. Moreover, for this class of antifungals, there is a third group of species that has shown in vitro intrinsic reduced susceptibility [60]. These three types of phenotypes have to be differentiated from low level-resistance and/or tolerance to echinocandins.

This section will be dedicated to summarizing what is known as clinical resistance mechanisms, thus the use of a cited bibliography is suggested to expand on the topic of tolerance and response to stress [44,46,54,55,56,57,58,59]. As a brief summary, we can state that the reduction of the β-1,3-D-glucan content of the cell wall leads to an important cellular stress that is sensed by Rho1p. In response, a complex network of stress response pathways and other compensatory pathways are activated, aiming to alter the qualitative composition of the cell wall to maintain its structural integrity. The main event is the increase of chitin synthesis that replaces in part the structural function of β-1,3-D-glucans, decreasing the cell sensitivity to echinocandins [116,117,118,119]. This shift on the relative composition of the cell wall is responsible (at least in part) for the paradoxical growth effect seen in vitro in several *Candida* spp. isolates when confronted with high echinocandin doses [120,121,122]. Other tolerance and reduced sensitivity to echinocandin mechanisms include changes in quantity and quality of membrane sphingolipids [123] and chromosomal instability (aneuploidy and other genomic rearrengements) [95,124,125]. As a conclusion, these mechanisms stabilize cells and soothe the effect of the drug against them, giving time for stable mechanisms to be selected [98].

The main mechanisms of echinocandin resistance, considered the universal mechanisms, are the *FKS* hot spot mutations [51,52,53,60,79,80,98]. These mechanisms are the main responsible factors for both intrinsic and secondary resistance to echinocandins with slight differences.

The association between echincandin treatment failures (secondary resistance) with the presence of a mutation in one (or both) of the hot spot regions of the Fksp is considered an independent risk factor for treatment failure for *C. glabrata* infections [96]. The identification of these mutations is better than MIC as a predictor of clinical response (specially for caspofungin MIC) [96] and as a predictor of the enzyme complex insensitivity to these drugs [51,52].

**Figure 4:**
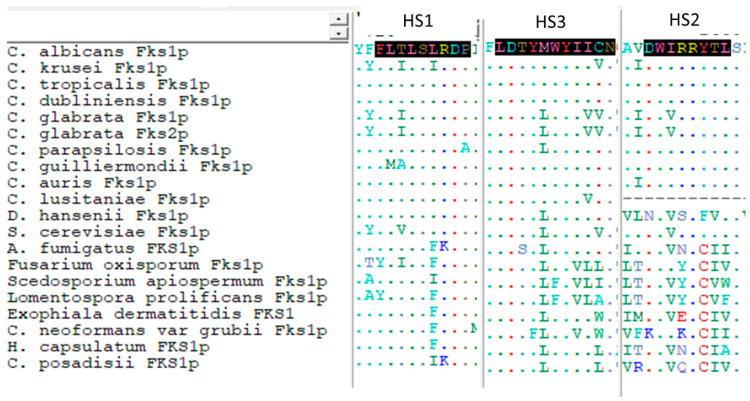
Clustal alignment of Fksps of different fungal pathogens. HS1: hot spot 1 region; HS2: hot spot 2 region; HS3: hot spot 3 region. Dots represent the same amino acid as *C. albicans* in its equivalent position.

Subtitutions in Fksp subunits conferring elevated MIC values are limited to two highly conserved amino acid regions named hot spot regions. Early reports consider that these regions should cover the amino acid residues between F641 and P649 (hot spot 1) and D1357 and L1364 (hot spot 2) (*C. albicans* Fks1p numbering following accession number # D88815) (Figure 3B and Figure 4) [51,52,53,99]. Later, it was suggested that hot spot 2 should be limited to only one amino acid (R1361 following the same aa. #) considering that this is the main amino acid of this hot spot that is linked with echinocandin resistance [98]. These mutations were described in the *FKS1* genes of diploid *Candida* spp. (most of the *Candida* spp. considered human pathogens, e.g., *C. albicans*, *C. tropicalis*, *C. krusei*) both in homo- and heterozygosity (they are dominant) [51,60,98,99,126,127,128,129,130]. In *C. glabrata* (haploid *Candida* spp.), echinocandin resistance was linked in *FKS1* and in *FKS2* (> prevalence for *FKS2* mutants) and it is the only species that would present mutations (or deletions) in both genes at the same time [52,70].

The described amino acid subtitutions reduce the sensitivity of glucan synthase to echinocandins between 30- to 3000-fold (IC_50_s), which translate into 10- to more than 100-fold MIC increase [51,52,60,70,98,99,127]. These resistance phenotypes have a cost to the fitness of the strains (confirmed with isogenic *C. albicans* strains using a competitive model of murine candidiasis). It was attributed to a reduction in the catalytic efficacy of the β-1.3-D-glucan synthase complex harboring mutant Fksp subunits (lower Vmax with no Km changes) that led to an altered cell wall composition [131]. As MIC and IC_50_ values, these Vmax reductions ranged between a 20% and 80% reduction for *C. albicans* and *C. glabrata FKS* mutants [51,52]. The described phenotype differences (Vmax/fitness) had a correlation with the prevalence of each of the different affected residues of the hot spots. Thus, in *C. albicans*, the substitutions at F641 and S645 (aa. #D88815) account for around 75–80% of the mutants. These mutants showed the lowest Vmax reduction with its consequent slight reduction in their fitness (when compared with other mutants). Similarly, in *C. glabrata*, the most common substitutions are at *FKS2*, but still are at equivalent positions than for *C. albicans* Fks1p (at its F659 and S663 residues, aa. # YLR342W). Unfortunately, this higher prevalence and relative virulence is also accompanied by a more marked resistance phenotype [51,52]. Amino acid substitutions in other positions of the hot spot confer no or less-pronounced phenotypes. These differences may be explained through considering the putative echinocandin-binding pocket model described before [91], together with the prediction that the hot spot regions are part of the protein arranged as an α-helix [132]. When these regions are represented as a helical wheel plot (or Edmundson wheel), the amino acid residues of the hot spot regions that are mainly linked with echinocandin resistance group together (Figure 3). These plots represent secondary structures with a helical potential arranging where the amino acids are drawn in a rotating manner with an angle of rotation of around 100° [94]. Thus, the amino acid sequence is represented as viewing the helix from above, showing whether certain amino acids are concentrated in one side of the helix or not. In the Fksp hot spot 1 region, the residues linked with echinocandin resistance (F641, S645, D648, and P649 following *C. albicans* numbering aa. # D88815) are concentrated in one side of the wheel. Similarly, the hot spot 2 residues linked with echinocandin resistance (W1358 and R1361) are also grouped together in the Edmundson wheel.

As the other echinocandin, rezafungin is inactive against *Fusarium* spp., *Scedosporium* spp., *Lomentospora prolificans*, Mucorales, *Histoplasma capsulatum*, and Basydiomycetes, including *Cryptococcus* spp., *Trichosporon* spp., etc. [60,98,133]. The mechanisms involved in these intrinsic resistances vary depending on the studied species. In the filamentous Ascomycetes *Fusarium* spp. and *L. prolificans*, the mechanism of resistance involves naturally occurring polimorfisms at the hot spot regions of the Fks1p. The implicated residues are located at F684Y, being equivalent to those related with high-level echinocandin clinical resistance in *C. albicans* (F641X) [134] (Figure 3B and Figure 4). For *Scedosporium* spp., the proposed mechanism involved is a polymorphism located in the so-called hot spot 3 region [89]. Although described in these pathogens and in *Saccharomyces cerevisiae* laboratory mutants [89], this region was not implicated in acquired clinical echincandin resistance [60,98]. For Mucorales, Ajellomycetaceae, and Basydiomycetes, the molecular mechanisms of their intrinsic echinocandin resistance is not clear. Some reports have suggested that β-1,3-D-glucans are not as important for cell wall integrity in these fungal pathogens in comparison to other fungi (lower content of β-1,3-D-glucans) [32]. Others suggest that *Cryptococcus* spp. and *Histoplasma capsulatum* melanization [135] and *Cryptococcus neoformans* liplid flippase subunit Cdc50 [93] may play a role in their reduced echinocandin susceptibility. Moreover, *Cryptococcus neoformans* β-1,3-D-glucan synthase complex was partially purified and its *FKS* subunits were sequenced. No mutations were observed, and the isolated enzyme complex was susceptible in vitro to echinocandins. These two results discard the implication of alterations in Fksp as a molecular mechanism of resistance in this species [65].

As mentioned, there are some *Candida* spp. that show naturally occurring polymorphisms at *FKS* hot spot regions. These species were crowded together within the intrinsic reduced echinocandin susceptibility (IRES) group that includes *C. parapsilosis* complex (*C. parapsilosis sensu stricto*, *C. metapsilosis*, and *C. orthopsilosis*), *C. guilliermondii*, etc. They showed higher echinocandin MIC values (>10-fold) and their glucan synthase complexes showed higher IC_50_s than the other *Candida* spp. The CLSI and EUCAST antifungal susceptibility testing committees propose higher breakpoints and epidemiological cut-off values to consider strains of these species as resistant or non-wild type, respectively [136,137,138]. Early clinical trials showed that the treatment of *C. parapsilosis* infections showed better results (clinical cure) when treated with fluconazole than with anidulafungin [139]. However, echinocandins are still the drug of choice to treat infections caused by IRES species [55]. Species with IRES phenotype show polymorphisms in residues located in the C-terminal end of the hot spot 1 region or in residues located in the opposite side of the helical wheel of the residues with prominent phenotype (they are grouped together) (Figure 3). This is the case for *C. parapsilosis* complex (polymorphism P649A, same numbering in *C. albicans* aa. # D88815) and for *C. guilliermondii* (L633M and T634A, equivalent to L642 and T643 of aa. # D88815) (Figure 4). The implication of these polymorphism in the IRES phenotype were molecularly confirmed [79,80]. Moreover, echinocandins need more time and higher doses to reduce the starting inocula by three logs (fungicidal threshold). This fact was confirmed using time-killing curves and minimal fungicidal concentration tests [77,81,140].

Turning to *Candida auris*, this species has some of the characteristics mentioned for species with IRES phenotype and other characteristics of the normally echinocandin-susceptible species. Epidemiological cut-off values for *C. auris* were firstly established for all echinocandins at 0.25 µg/mL (using Indian isolates) [141]. Later, the U.S. Center for Disease Control and Prevention (CDC) proposed tentative breakpoints on the basis of the MIC modal distributions of more than 100 strains from different geographic locations (anidulafungin ≥ 4 µg/mL, caspofungin ≥ 2 µg/mL, and micafungin ≥ 4 µg/mL) [85]. These breakpoints are very similar to those for *C. parapsilosis* (anidulafungin ≥ 8 µg/mL and micafungin ≥ 4 µg/mL) [138]. Killing kinetics assays demonstrated that *C. auris* is tolerant to echinocandins and that there were no fungicidal activity against this species [83]. On the other hand, *C. auris* shows a “wild type” hot spot region (FLTLSLRDP)) (Figure 4), and echinocandin resistance was related to acquired substitution post-echinocandin treatment [81,130,142].

Turning to rezafungin and considering that it is an anidulafungin analog with extended half-life, it was thought from the start that it would be likely to develop resistance. Locke et al. characterized the in vitro resistance development of four *Candida* spp. (*C. albicans*, *C. glabrata*, *C. krusei*, and *C. parapsilosis*) using two different mutant selection methods (spontaneous resistance and resistance after successive passages) and using anidulafungin and caspofungin as comparators [143]. The selection of spontaneous mutants (single-step/high-dose method) occurred at very low frequencies for all the three drugs. Mutations at *FKS* occurred at similar ranges for rezafungin than for anidulafungin and caspofungin (1 mutant per every 1.5 × 10^8^ cells, per 1.6 × 10^7^–3.9 × 10^9^ cells, and 3.5 × 10^7^–3.9 × 10^9^ cells, respectively) [144]. As for the other echinocandins, rezafungin selected *FKS* mutants easier in *C. glabrata* (haploid) than in *C. albicans*, while *C. parapsilosis* and *C. krusei* seemed to have lower potential of *FKS*-linked resistance development [130,144,145]. Moreover, cross-resistance was observed in *FKS* mutants independently of the used echinocandin for mutant selection. When using successive passages, 20 passages were needed to obtain strains with increased MIC values. However, most of the mutants selected by passages showed no *FKS* mutations and those with no hot spot substitutions showed slight MIC increases (2–4-fold) [144]. When rezafungin was used as a selector of spontaneous mutants, only the so-called strong phenotype mutations (which were the most prevalent in clinical settings) were obtained, including S645P in *C. albicans* Fks1p and F659Δ and S663F in *C. glabrata* Fks2p [60,98,144,146]. Less common or barely described substitutions were also encountered in *C. glabrata* Fks2p as R665G, D666H, D666N, D666Y, and R1378S (the latter in hot spot 2) [144,147] (Figure 3). Similar substitutions were obtained when serial passages were used to select mutants. Some were commonly described in clinical isolates, such as S645Y in Fks1p of *C. albicans*, and D632Y and F659I in *C. glabrata* Fks1p and Fks2p, respectively. As with spontaneous mutant selection, the same “rare” substitutions were found, such as D666N and D666Y in *C. glabrata* Fks2p and the never previously described I1366S in the hot spot 2 of the Fks1p of *C. krusei* [144].

Zhao et al. studied the kinetic inhibition (IC_50_) of partially purified *C. albicans* β-1,3-D-glucan synthases by micafungin and rezafungin. They found that both echinocandins were able to inhibit wild type enzyme complexes at very low concentrations (around 15 ng/mL for both drugs). However, rezafungin was shown to be a better inhibitor of mutant complexes harboring Fks1p subunits with F641S substitutions in comparison with micafungin (24-fold vs. 100-fold increase in IC_50_ values when compared with wild type enzyme complexes). On the other hand, enzyme complexes with homozygous S645P mutation showed similar IC_50_ values for both echinocandins (>140-fold increase) [100]. When *C. glabrata* was studied, the semi-purified enzyme complexes harboring Fks2p subunits with F659Δ deletion was very resistant to both echinocandins (IC_50_ > 10,000 ng/mL), while the one harboring the S663P mutation at Fks2p was more resistant to rezafungin than for micafungin [100].

The same group of researchers compared the mutant prevention concentration of these two echinocandins [100]. This concept was coined for bacteriology at the beginning of this century and is defined as the concentration above which it is possible to inhibit the population of strains able to persist at concentrations above the MIC and that are the source of future mutants with stable resistance phenotypes. Thus, if this concentration is reached, the selection of mutants is less likely [148]. For rezafungin and micafungin, the mutant prevention concentration was 16 µg/mL for *C. albicans* and *C. glabrata* for both drugs. Taking all these data together, it seems that rezafungin has a low potential of resistance development that is comparable to other echinocandins. However, rezafungin showed a better tissue penetration [149] and better PK/PD pharmacometrics [150,151]. These facts would allow rezafungin to reach higher tissue concentrations, surpassing the mutation selection threshold.

## 9. Conclusions

- Rezafungin is a new β-glucan synthase inhibitor that is chemically related to anidulafungin, with better stability, tissue penetration, and PK/PD pharmacometrics.
- Rezafungin shares targets and mechanisms of action with the other echinocandins but with an improved safety profile, allowing the potential administration of higher doses.
- Rezafungin is much more stable in solution than the older echinocandins. This fact is beneficial in terms of dosing flexibility and storage but also in terms of manufacturing.
- Rezafungin is proposed as a drug to be administered once-weekly (intravenous). New forms of administration are in development such as topical and subcutaneous forms.
- Higher dose regimens will eventually prevent (or reduce) the selection of resistant strains.
- Rezafungin MIC_50_s against *Candida* spp. mimicked those of anidulafungin. It is very potent in terms of in vitro activity against *Aspergillus* spp. (including multidrug-resistant cryptic species). Still, its spectrum is narrow. Basidiomycetes, Mucorales, *Fusarium* spp., and Ajellomycetaceae are intrinsically resistant to rezafungin.
- Rezafungin shares with the older echinocandins the same target and the same mechanisms of resistance. The selection of FKS mutants occurred at a similar frequency for rezafungin than for anidulafungin and caspofungin.
- When rezafungin begins to be used clinically, the appearance of described rare mutations in *C. glabrata* Fks2p (R665G, D666H, D666N, D666Y, and R1378S) should be monitored as they may be a unique resistance mechanism for this drug. This monitoring will be essential to be able to adapt the few molecular diagnostic methods of echinocandin resistance that have been developed.

## Figures and Tables

**Table 1:** Rezafungin and FDA-approved echinocandin MIC values for *Candida* spp. with wild type *FKS* genes determined by Clinical and Laboratory Standards Institute (CLSI) and/or European Committee on Antimicrobial Susceptibility Testing (EUCAST) microdilution reference methods.

*Candida* spp.	*n*	RZF ^a^		ANF ^a^		CSF ^a^		MCF ^a^		References
		MIC_50_	MIC_90_	MIC_50_	MIC_90_	MIC_50_	MIC_90_	MIC_50_	MIC_90_	
*C. albicans*	2612	0.022	0.050	0.012	0.027	0.053	0.069	0.022	0.021	[100,101,102,103,104,105,106,107,109,110,111]
*C. glabrata*	1541	0.044	0.085	0.045	0.085	0.080	0.140	0.027	0.030	[100,101,102,103,104,105,106,107,109,110,111]
*C. krusei*	773	0.033	0.078	0.045	0.085	0.280	0.248	0.108	0.153	[100,101,102,103,104,105,106,107,109,110,111]
*C. parapsilosis sensu stricto*	1156	1.260	2.000	1.219	2.245	0.435	0.758	1.122	1.414	[100,101,102,103,104,105,106,107,109,110,111]
*C. tropicalis*	959	0.030	0.072	0.012	0.034	0.046	0.092	0.030	0.050	[100,101,102,103,104,105,106,107,109,110,111]
*C. dubliniensis*	207	0.060	1.360	0.034	0.270	0.036	0.370	0.030	0.105	[100,101,102,103,104,105,107,109,110]
*C. auris*	237	0.153	0.500	0.391	0.250	0.707	1.000	0.630	0.500	[105,107,109,110]
*C. lusitaniae*	66	0.120	0.250	0.042	0.060	0.500	1.000	0.038	0.250	[107,110]
*C. kefyr*	52	0.06	0.12	0.03	0.06	0.25	0.50	0.06	0.12	[107]
*C. guilliermondii*	27	1.00	1.00	1.00	2.00	0.50	1.00	1.00	2.00	[107]
*C. orthopsilosis*	25	0.500	1.000	0.707	1.000	0.354	0.707	0.500	1.000	[107,109]
*C. metapsilosis*	15	0.50	0.50	0.25	0.50	0.25	0.50	0.25	0.50	[107]
*C. fabianii*	15	0.06	0.12	0.06	0.12	1.00	1.00	0.06	0.12	[107]
*C. insconspicua*	41	0.06	0.06	0.008	0.015	0.25	0.50	0.03	0.06	[107]
*C. sojae*	10	0.06	0.06	0.015	0.03	0.25	0.50	0.03	0.06	[107]
*C. lipolytica*	10	0.06	0.06	0.06	0.12	0.25	0.50	0.25	1.00	[107]
*C. pulcherrima*	10	0.03	0.06	0.015	0.06	0.50	1.00	0.06	0.25	[107]

RZF: rezafungin, ANF: anidulafungin, CSF: caspofungin, MCF: micafungin. ^a^ Geometric means of the data published in the cited references expressed in µg/mL. ND: no data available.

**Table 2:** Rezafungin and FDA-approved echinocandin MIC values for *Candida* spp. with mutant *FKS* genes determined by CLSI microdilution reference methods.

*Candida* spp. *FKS* Mutants	*n*	RZF ^a^		ANF ^a^		CSF ^a^		MCF ^a^		References
		MIC_50_	MIC_90_	MIC_50_	MIC_90_	MIC_50_	MIC_90_	MIC_50_	MIC_90_	
*C. albicans*	20	0.71	1.00	0.50	1.00	0.50	1.00	1.00	ND	[100,104]
*C. glabrata*	21	0.50	1.00	0.25	1.00	0.50	1.00	1.00	ND	[100,104]
*C. krusei*	6	0.35	1.00	0.50	2.00	1.00	16.00	1.00	ND	[100,104]
*C. tropicalis*	9	0.71	1.00	0.50	1.00	1.00	2.00	2.00	ND	[100,104]
*C. dubliniensis*	1	0.03	ND	ND	ND	ND	ND	0.03	ND	[100]
*C. auris*	4	8.00	8.00	8.00	ND	4.00	ND	4.00	ND	[105]

RZF: rezafungin, ANF: anidulafungin, CSF: caspofungin, MCF: micafungin. ^a^ Geometric means of the data published in the cited references expressed in µg/mL. ND: no data available.

**Table 3:** Rezafungin and FDA-approved echinocandin MEC values for *Aspergillus* spp. determined by CLSI microdilution reference methods.

*Aspergillus* spp.	*n*	RZF ^a,b^		ANF ^a,b^		CSF ^a,b^		MCF ^a,b^		References
		MEC_50_	MEC_90_	MEC_50_	MEC_90_	MEC_50_	MEC_90_	MEC_50_	MEC_90_	
*A. fumigatus sensu stricto*	305	0.018	0.025	0.010	0.019	0.036	0.050	0.009	0.015	[101,102,104,109]
*A. calidoustus*	11	0.060	0.060	ND	ND	0.120	4.000	0.008	0.030	[101]
*A. lentulus*	11	0.080	0.080	ND	ND	0.060	0.250	0.008	0.030	[101]
*A. thermomutatus*	5	0.060	ND	ND	ND	0.060	ND	0.030	ND	[101]
*A. udagawae*	5	0.015	ND	ND	ND	0.060	ND	0.008	ND	[101]
*A. section Terrei*	19	0.015	0.015	0.015	0.015	0.120	0.250	ND	ND	[104]
*A. flavus*	12	0.015	0.015	0.015	0.015	0.120	0.250	ND	ND	[104]
*A. section Flavii*	57	0.004	0.015	0.004	0.015	0.015	0.030	0.015	0.030	[102]
*A. niger*	16	0.008	0.030	0.008	0.008	0.060	0.120	ND	ND	[104]

RZF: rezafungin, ANF: anidulafungin, CSF: caspofungin, MCF: micafungin. ^a^ Geometric means of the data published in the cited references expressed in µg/mL. ND: no data available. ^b^ Susceptibility shown are minimal effective concentration (MEC) values.

## Supplementary Materials

The following are available online at https://www.mdpi.com/2309-608X/6/4/262/s1, Table S1: In vitro data for rezafungin and the other echinocandins used ass comparators for *Candida* spp. and *Aspergillus* spp. determined by CLSI and EUCAST susceptibility testing method.
