# Supplementary material for: Rezafungin—Mechanisms of Action, Susceptibility and Resistance: Similarities and Differences with the Other Echinocandins
Source: J Fungi (Basel). 2020 Nov 1;6(4):262. doi: 10.3390/jof6040262 (PMC7711656; doi:10.3390/jof6040262)
Supplement: Supplementary file 1 [file jof-06-00262-s001.pdf]

Table S1: In vitro data for rezafungin and the other echinocandins used ass comparators for Candida spp. and Aspergillus spp. determined by CLSI and EUCAST susceptibility testing method.

Species	n	RZF[a,b]		ANF[a,b]		CSF[a,b]		MCF[a,b]		AST method	Type of study	Other data	Ref.
		MIC_{50}	MIC_{90}	MIC_{50}	MIC_{90}	MIC_{50}	MIC_{90}	MIC_{50}	MIC_{90}				
C. albicans	403	0.015	0.03							EUCAST	Multicenter	WT/UL [a]: 0.06	[106]
	100	0.008	0.03	0.004	0.008					CLSI	One center US strains		[104]
	569	0.06	0.06	0.003-0.25				0.03-2.00		EUCAST	Multicenter	WT/UL [a]: 0.12 (Northern Europe). 1.1% >WT-UL	[108]
	125	0.03	0.06	0.006	0.03	0.25	0.50	0.015	0.015	CLSI	One UK center - Reference lab.	29.6% of I to CSF	[105]
	304	0.03	0.06	0.015	0.03	<0.06	0.015	<0.08	0.015	CLSI	One center – Reference lab – worldwide isolates		[101]

	10	<0.03 (<0.03)						<0.03 (<0.03)		CLSI	One lab – Strain collection	FKS sequenced.	[98]
	251	0.03	0.06	0.015	0.06	0.03	0.06	0.015	0.03	CLSI	One center – Reference lab – worldwide isolates		[107]
	15	0.015	0.06	0.015	0.03	0.12	0.12			CLSI	One center – Reference lab – worldwide isolates	FKS genes sequenced	[102]
	835	0.03	0.06	0.015	0.03	0.015	0.03	0.015	0.03	CLSI	One center – Reference lab – worldwide isolates (isolated between 2016-2018)		[100]
C. albicans FKS mutant	10	0.25	1.00	0.50	1.00	0.50	1.00			CLSI	One center – Reference lab – worldwide isolates	FKS genes sequenced	[102]
	10	MIC_{50}= 2.00 (0.12-2.00)						MIC_{50} = 1.00 (0.03-4.00)		CLSI	One lab – Strain collection	FKS genes sequenced. Strains	[98]

												classified by its CSF susceptibility/resistance	
C. glabrata	413	0.031 - 0.125	0.12							EUCAST	Multicenter	WT/UL [a]: 0.12	[106]
	100	0.03	0.03	0.03	0.03					CLSI	One center US strains		[104]
	328	0.12	0.12	0.006-1.00 (3.3%> ECOFF)				0.06-0.50 (4.9% > ECOFF		EUCAST	Multicenter	WT/UL [a]: 0.25 (all strains from Northern Europe). 3%>WT-UL	[108]
	81	0.06	0.12	0.03	0.06	0.50	0.50	0.03	0.06	CLSI	One UK center - Reference lab.	32.1% and	[105]

												63% of the strains were classified as intermediate and resistant to CSF, respectively	
	121	0.03	0.12	0.06	0.12	0.03	0.06	<0.06	0.015	CLSI	One center – Reference lab – worldwide isolates		[101]
	9	MIC_{50}=0.06 (<0.03-0.06)						MIC_{50}=<0.03 (<0.03)		CLSI	One lab – Strain collection	FKS genes sequenced. Strains classified by its	[98]

												CSF susceptibility/resistance	
	100	0.03	0.06	0.06	0.12	0.06	0.12	0.015	0.03	CLSI	One center – Reference lab – worldwide isolates		[107]
	15	0.06	0.06	0.06	0.12	0.12	0.25			CLSI	One center – Reference lab – worldwide isolates	FKS genes sequenced	[102]
	374	0.016	0.12	0.016	0.12	0.03	0.06	0.015	0.03	CLSI	One center – Reference lab – worldwide isolates (isolated between 2016-2018)		[100]
C. glabrata FKS mutants	10	0.25	1.00	0.25	1.00	0.50	1.00			CLSI	One center – Reference lab – worldwide isolates	FKS genes sequenced	[102]
	11	MIC_{50}= 1.00						(0.06-4.00)[d]		CLSI	One lab – Strain collection	FKS genes sequen	[98]

		(0.12-4.00)										ced. Strains classified by its CSF susceptibility/resistance	
C. krusei	402	0.06	0.12							EUCAST	Multicenter	WT/UL[a]: 0.12	[106]
	100	0.008	0.06	0.03	0.03					CLSI	One center US and Russian strains		[104]
	82	0.12	0.12	0.015-0.25 (2.4%> ECOFF)				0.06-4 (3.7% > ECOFF)		EUCAST	Multicenter	WT/UL[a]: 0.25 (all strains from Northern Europe). 1.2% >WT/UL	[108]

	53	0.06	0.12	0.06	0.12	1.00	1.00	0.25	0.25	CLSI	One UK center - Reference lab.	22.6% and 66.1% of the strains were intermediate and resistant to CSF, respectively	[105]
	14	0.03	0.06	0.03	0.12	0.12	0.25	0.06	0.12	CLSI	One center – Reference lab – worldwide isolates		[101]
	11	MIC_{50}=<0.03 (<0.03-0-06)						MIC_{50}=0.12 (0.03-0.25)		CLSI	One lab – Strain collection	FKS genes sequenced. Strains classified by its	[98]

												CSF susceptibility/resistance	
	18	0.03	0.06	0.06	0.12	0.25	0.25			CLSI	One center – Reference lab – worldwide isolates	FKS genes sequenced	[102]
	77	0.03	0.06	0.06	0.12	0.25	0.06	0.12		CLSI	One center – Reference lab – worldwide isolates (isolated between 2016-2018)		[100]
	16	0.03	0.06	0.06	0.06	0.23	0.25	0.06	0.12	CLSI	One center – Reference lab – worldwide isolates		[107]
C. krusei FKS mutants	4	0.03 (0.03-8.00)						<0.03-4.00[d]		CLSI	One lab – Strain collection	FKS genes sequenced. Strains classified by its	[98]

												CSF susceptibility/resistance	
	2	0.25	1.00	0.50	2.00	1.00	>8.00			CLSI	One center – Reference lab – worldwide isolates	FKS genes sequenced	[102]
C. parapsilosis sensu lato	398	2.00	2.00							EUCAST	Multicenter	WT/UL[a]: 4.00	[106]
	100	1.00	1.00	0.50	2.00					CLSI	One center US strains		[104]
	61	2.00	4.00	0.25-2.00 (0%>ECOFF)				0.25-4.00 (0%>ECOFF)		EUCAST	Multicenter	WT/UL[a]: 4.00 (all strains from Northern Europe). 0%>wT/UL	[108]

	59	1.00	2.00	1.00	2.00	1.00	2.00	1.00	2.00	CLSI	One UK center - Reference lab.	No resistance	[105]
	83	1.00	2.00	2.00	2.00	0.25	0.50	1.00	1.00	CLSI	One center – Reference lab – worldwide isolates		[101]
	19	MIC$_{50}$=2.00(2.00-4.00)						MIC$_{50}$=4.00(2.00-8.00)		CLSI	One lab – Strain collection	FKS genes sequenced. Strains classified by its CSF susceptibility/resistance	[98]
	92	1.00	2.00	2.00	4.00	0.50	1.00	1.00	2.00	CLSI	One center – Reference lab – worldwide isolates		[107]
	15	1.00	2.00	2.00	2.00	0.50	0.50			CLSI	One center – Reference lab – worldwide isolates	FKS genes	[102]

												sequenced	
	329	1.00	2.00	2.00	2.00	0.25	0.50	1.00	1.00	CLSI	One center – Reference lab – worldwide isolates (isolated between 2016-2018)		[100]
C. tropicalis	402	0.03	0.12							EUCAST	Multicenter	WT/UL[a]: 0.25	[106]
	100	0.008	0.03	0.004	0.015					CLSI			[104]
	73	0.12	0.25	0.003—2 (2.7%> ECOFF)				0.015-2.00 (2.7%>ECOFF)		EUCAST	Multicenter	WT/UL[a]: 0.25 (all strains from Northern Europe). 2.7%> WT/UL	[108]
	51	0.06	0.06	0.015	0.03	0.25	0.50	0.03	0.06	CLSI	One UK center - Reference lab.	29.4% of the strains	[105]

												were considered intermediate to CSF	
	55	0.03	0.06	0.015	0.03	0.015	0.03	0.015	0.03	CLSI	One center – Reference lab – worldwide isolates		[101]
	16	0.03	0.06	0.03	0.06	0.12	0.12			CLSI	One center – Reference lab – worldwide isolates	FKS genes sequenced	[102]
	196	0.03	0.06	0.03	0.06	0.015	0.06	0.03	0.06	CLSI	One center – Reference lab – worldwide isolates (isolated between 2016-2018)		[100]
	15	MIC_{50}=0.03 (0.03)						MIC_{50}=0.03 (0.03-0.03)		CLSI	One lab – Strain collection	FKS genes sequenced. Strains classifie	[98]

												d by its CSF susceptibility/resistance	
	51	0.015	0.06	0.015	0.03	0.03	0.06	0.03	0.06	CLSI	One center – Reference lab – worldwide isolates		[107]
C. tropicalis FKS mutants	5	0.25	1.00	0.50	1.00	1.00	2.00			CLSI	One center – Reference lab – worldwide isolates	FKS genes sequenced	[102]
	4	MIC_{50}=2.00 (0.25-2.00)						MIC_{50}=2.00(1.00-2.00)		CLSI	One lab – Strain collection	FKS genes sequenced. Strains classified by its CSF susceptibility/r	[98]

												esistance	
C. dubliniensis	1	0.03						0.03		CLSI	One lab – Strain collection	FKS genes sequenced. Strains classified by its CSF susceptibility/resistance	[98]
	68	0.06	0.12	0.003-0.25 (2.9%>ECOFF)				0.06-2.00 (2.9%>ECOFF)		EUCAST	Multicenter	WT/UL[a]: 0.25 (all strains from Northern Europe). 2.9%	[108]

												> WT/UL	
	22	0.50	1.00	0.015	0.03	0.12	0.25	0.03	0.03	CLSI	One UK center - Reference lab.		[105]
	12	0.03	0.06	0.03	0.06	0.015	0.03	0.015	0.015	CLSI	One center – Reference lab – worldwide isolates		[101]
	11	0.03	0.06	0.06	0.06	0.03	0.06	0.03	0.03	CLSI	One center – Reference lab – worldwide isolates		[107]
	93	0.06	0.12	0.03	0.12	0.03	0.03	0.03	0.03	CLSI	One center – Reference lab – worldwide isolates (isolated between 2016-2018)		[100]
C. dubliniensis FKS mutant	1	0.03						0.03		CLSI	One lab – Strain collection	FKS genes sequenced. Strains classified by its CSF suscept	[98]

												ibility/resistance	
C. auris	96	0.12	0.50	1->16		0.5->16		0.5->8		CLSI	One center using worldwide isolates	Some strains showed high echinocandin MIC with no FKS mutations. These strains showed low RZF MIC.	[103]
	122	0.25	1.00	0.016->32 (27%>ECOFF)				0.03->32 (6.6%>ECOFF)		EUCAST	Multicenter	WT/UL [a]: 0.50 (all isolates	[108]

												from India). 14.8%> WT/UL	
	19	0.12	0.25	0.06	0.25	0.50	1.00	0.25	0.50	CLSI	One UK center - Reference lab.		[105]
C. auris (S639P)	4	8	8	8		1->16		4-8					[103]
C. lusitaniae	20	0.12	0.25	0.03-0.25 (5%>ECOFF)				0.03-0.50 (5%>ECOFF)		EUCAST	Multicenter	WT/UL [a]: 0.25 (all strains from Northern Europe). 0%>WT/UL	[108]
	46	0.12	0.25	0.03	0.06	0.5	1.00	0.12	0.25	CLSI	One UK center - Reference lab.		[105]
C. kefyr	52	0.06	0.12	0.03	0.06	0.25	0.50	0.06	0.12	CLSI	One UK center - Reference lab.		[105]
C. guilliermondii	27	1.00	1.00	1.00	2.00	0.50	1.00	1.00	2.00	CLSI	One UK center - Reference lab.		[105]

C. orthopsilosis	15	0.50	1.00	1.00	1.00	0.50	1.00	0.50	1.00	CLSI	One UK center - Reference lab.		[105]
	10	0.50	1.00	0.50	1.00	0.25	0.50	0.50	1.00	CLSI	One center – Reference lab – worldwide isolates		[107]
C. metapsilosis	15	0.50	0.50	0.25	0.50	0.25	0.50	0.25	0.50	CLSI	One UK center - Reference lab.		[105]
C. fagianii	15	0.06	0.12	0.06	0.12	1.00	1.00	0.06	0.12	CLSI	One UK center - Reference lab.		[105]
C. insconspicua	41	0.06	0.06	0.008	0.015	0.25	0.50	0.03	0.06	CLSI	One UK center - Reference lab.		[105]
C. sojae	10	0.06	0.06	0.015	0.03	0.25	0.50	0.03	0.06	CLSI	One UK center - Reference lab.		[105]
C. lipolytica	10	0.06	0.06	0.06	0.12	0.25	0.50	0.25	1.00	CLSI	One UK center - Reference lab.		[105]
C. pulcherrima	10	0.03	0.06	0.015	0.06	0.50	1.00	0.06	0.25	CLSI	One UK center - Reference lab.		[105]
Aspergillus fumigatus [c]	56	0.015	0.015	<0.008	0.015	0.03	0.03	0.015	0.015	CLSI	One center – Reference lab – worldwide isolates		[107]
	20	0.015	0.015	0.015	0.015	0.06	0.12			CLSI	One center – Reference lab – worldwide isolates		[102]

	183	0.015	0.03	0.015	0.03	0.015	0.03	<0.008	0.015	CLSI	One center – Reference lab – worldwide isolates		[100]
Aspergillus fumigatus sensu stricto [c]	46	<0.015/0.06 (Azole S/Azole R)	0.06/0.12			0.03/0.06	0.03/0.06	<0.015/<0.015	<0.015/0.06	CLSI	One US center	31 azole resistant (VRC MIC90 = >16.00 μg/ml)	[99]
Aspergillus calidoustus [c]	11	0.06	0.06			0.12	4.00	<0.015	0.03	CLSI	One US center	VRC MIC90 = 4.00 μg/ml	155)
Aspergillus lentulus [c]	11	<0.015	<0.015			0.06	0.25	<0.015	0.03	CLSI	One US center	VRC MIC90 = 8.00 μg/ml	155)
Aspergillus thermomutatus [c]	5	(<0.015-0.25)				(0.03-0.25)		(<0.015-0.06)		CLSI	One US center	VRC MIC range 1-8.00 μg/ml	155)
Aspergillus udagawae [c]	5	(<0.015-0.03)				(0.03-0.12)		(<0.015)		CLSI	One US center	VRC MIC	155)

												range 0.50-2.00 µg/ml	
Aspergillus terreus [c]	19	0.015	0.015	0.015	0.015	0.12	0.25			CLSI	One center – Reference lab – worldwide isolates		[102]
Aspergillus flavus [c]	12	0.015	0.015	0.015	0.015	0.12	0.25			CLSI	One center – Reference lab – worldwide isolates		[102]
Aspergillus section flavii	45	<0.008	0.015	<0.008	0.015	0.015	0.03	0.015	0.03	CLSI	One center – Reference lab – worldwide isolates		[100]
Aspergillus niger [c]	16	<0.015	0.03	<0.015	<0.015	0.06	0.12			CLSI	One center – Reference lab – worldwide isolates		[102]

[a] Expressed in µg/ml. WT/UL: Wild type upper limit value. [b] MIC50 and MIC90 data if available if not ranges in parenthesis. [c] Susceptibility shown are Minimal effective concentration (MEC) values. [d] No MIC50 was included in the original paper.

RZF: rezafungin, ANF: anidulafungin, CSF: caspofungin, MCF: micafungin, VRC: voriconazole.
